# Supplementary material for: Determinants of Accepting or Rejecting Influenza Vaccination—Results of a Survey Among Ligurian Pharmacy Visitors During the 2023/2024 Vaccination Campaign
Source: Vaccines (Basel). 2025 May 29;13(6):580. doi: 10.3390/vaccines13060580 (PMC12197350; doi:10.3390/vaccines13060580)
Supplement: Supplementary file 1 [file vaccines-13-00580-s001.zip › vaccines-3610952-supplementary.pdf]

Table S1. Factors associated with flu vaccine intent or uptake in the 2023/24 season. Results of univariate and multiple logistic regression with odds ratios (OR), 95% confidence intervals (CI) and p-values reported.

Factors		No	Yes	Univariate			Multivariate		
				OR	(95%CI)	p value	OR	(95%CI)	p value
Sex	Female	6307 (37.9%)	10355 (62.1%)	Ref.			Ref.		
	Male	5098 (36.8%)	8739 (63.2%)	1.04	0.98 - 1.09	0.070	0.95	0.86 - 1.04	0.263
Age		49.4 ± 16.27	66.6 ± 15.76	1.06	1.06 - 1.07	<0.001	1.04	1.03 - 1.04	<0.001*
Education	Elementary school	358 (14.3%)	2143 (85.7%)	Ref.			Ref.		
	Middle school	1565 (27.4%)	4154 (72.6%)	0.44	0.39 - 0.50	<0.001	0.84	0.66- 1.06	0.148
	High school (2-3 years)	1366 (35.5%)	2483 (64.5%)	0.30	0.27 - 0.34	<0.001	0.84	0.66 - 1.08	0.184
	High school (4-5 years)	4543 (43.6%)	5868 (56.4%)	0.22	0.19 - 0.24	<0.001	0.91	0.72 - 1.14	0.409
	University degree	3573 (44.6%)	4446 (55.4%)	0.21	0.18 - 0.23	<0.001	1.01	0.80- 1.28	0.902
Over the past 12 months, how often do you see your GP?	Never	1033 (61.0%)	660 (39.0%)	Ref.				Ref.	
	Rarely	3487 (57.4%)	2593 (42.6%)	1.16	1.04 - 1.30	0.007	1.20	0.98 - 1.48	0.080
	Sometimes	3527 (37.7%)	5819 (62.3%)	2.58	2.32 - 2.87	<0.001	1.54	1.26 - 1.89	<0.001*
	Often	1444 (21.9%)	5136 (78.1%)	5.56	4.97 - 6.24	<0.001	1.97	1.59 - 2.44	<0.001*
	Very often	684 (19.7%)	2795 (80.3%)	6.37	5.63 - 7.28	<0.001	1.98	1.55 - 2.53	<0.001*
Over the past 12 months, how often do you go to the pharmacy?	Never	457 (59.9%)	306 (40.1%)	Ref.				Ref.	
	Rarely	1262 (58.1%)	909 (41.9%)	1.08	0.91- 1.27	0.395	0.91	0.67 - 1.25	0.572
	Sometimes	3405 (48.7%)	3586 (51.3%)	1.57	1.35 -1.83	<0.001	1.10	0.83 - 1.46	0.492
	Often	2993 (33.1%)	6061 (66.9%)	3.02	2.60 - 3.52	<0.001	1.13	0.86 - 1.50	0.384
	Very often	1992 (24.9%)	5995 (75.1%)	4.49	3.86- 5.24	<0.001	1.11	0.84- 1.49	0.444
In the past 12 months, was it easy for you or a family member	Very difficult	959 (39.5%)	1467 (60.5%)	Ref.				Ref.	
	Somewhat difficult	2099 (32.3%)	4409 (67.7%)	1.37	1.25 - 1.51	<0.001	1.00	0.83 - 1.21	0.973
	Neither easy nor difficult	4586 (40.2%)	6830 (59.8%)	0.97	0.89 - 1.06	0.558	1.00	0.84 - 1.20	0.961
	Somewhat easy	2745 (37.6%)	4563 (62.4%)	1.09	0.99 - 1.19	0.084	0.83	0.69 - 1.00	0.053

contact healthcare professionals (e.g., family doctor, specialist, nurses, etc.)?	Very easy	1016 (35.8%)	1825 (64.2%)	1.17	1.05 - 1.31	0.005	0.91	0.73 - 1.13	0.414
Covid-19 Vaccine	No	2488 (75.6%)	802 (24.4%)	Ref.				Ref.	
	Yes	8917 (32.8%)	18292 (67.2%)	6.36	5.857 - 6.922;	<0.001	3.07	2.635 - 3.578	<0.001*
Flu Vaccine 2022/23 season	No	10346 (86.5%)	1620 (13.5%)	Ref.				Ref.	
	Yes	1059 (5.7%)	17474 (94.3%)	105.38	97.21 - 114.34	<0.001	71.73	65.38 - 78.78	<0.001*

Table S2. Factors associated with reasons for not receiving the influenza vaccine. Results of univariate regression with odds ratios (OR), 95% confidence intervals (CI), p-values and Bonferroni-adjusted p-value reported.

**Univariate regression**																
		**Reason1**			**Reason2**			**Reason3**			**Reason4**			**Reason5**		
**Predictor**		**OR 95%CI**	**p raw**	**P Bonf**	**OR 95%CI**	**p raw**	**P Bonf**	**OR 95%CI**	**p raw**	**P Bonf**	**OR 95%CI**	**p raw**	**P Bonf**	**OR 95%CI**	**p raw**	**P Bonf**
**Sex**	**Male vs Female**	**1.08 (0.98–1.19)**	**0.128**	**0.99**	**0.89 (0.82–0.97)**	**0.008**	**0.776**	**1.10 (0.97–1.25)**	**0.134**	**0.99**	**1.03 (0.89–1.19)**	**0.700**	**0.99**	**0.89 (0.83–0.96)**	**0.004**	**0.381**
**Age (decade)**		**0.65 (0.63 – 0.67)**	**<0.001**	**<0.001**	**0.73 (0.71 – 0.74)**	**<0.001**	**<0.001**	**0.77 (0.75 – 0.80)**	**<0.001**	**<0.001**	**0.73 (0.70 – 0.75)**	**<0.001**	**<0.001**	**0.63 (0.61 – 0.64)**	**<0.001**	**<0.001**
**Education**	**Elementary school**	**Ref.**			**Ref.**			**Ref.**			**Ref.**			**Ref.**		
	**Middle school**	**3.54 (2.39–5.48)**	**<0.001**	**<0.001**	**1.46 (1.17–1.83)**	**0.001**	**0.098**	**1.70 (1.23–2.41)**	**0.002**	**0.204**	**1.37 (0.92–2.10)**	**0.130**	**0.99**	**3.13 (2.36–4.22)**	**<0.001**	**<0.001**
	**High school (2-3 years)**	**4.35 (2.91–6.78)**	**<0.001**	**<0.001**	**2.22 (1.78–2.80)**	**<0.001**	**<0.001**	**2.73 (1.97–3.88)**	**<0.001**	**<0.001**	**1.79 (1.18–2.77)**	**0.007**	**0.711**	**4.41 (3.32–5.97)**	**<0.001**	**<0.001**
	**High school (4-5 years)**	**7.40 (5.10–11.25)**	**<0.001**	**<0.001**	**2.39 (1.96–2.95)**	**<0.001**	**<0.001**	**2.31 (1.71–3.21)**	**<0.001**	**<0.001**	**2.65 (1.86–3.92)**	**<0.001**	**<0.001**	**6.32 (4.85–8.41)**	**<0.001**	**<0.001**
	**University degree**	**7.46 (5.13–11.36)**	**<0.001**	**<0.001**	**2.01 (1.63–2.49)**	**<0.001**	**<0.001**	**1.68 (1.23–2.36)**	**0.002**	**0.177**	**2.54 (1.77–3.77)**	**<0.001**	**<0.001**	**7.93 (6.09–10.56)**	**<0.001**	**<0.001**
**MMG**	**Never**	**Ref.**			**Ref.**			**Ref.**			**Ref.**			**Ref.**		

	Rarely	1.00 (0.84–1.19)	0.975	0.99	0.87 (0.75–1.02)	0.091	0.99	0.93 (0.75–1.17)	0.539	0.99	0.92 (0.70–1.21)	0.526	0.99	0.90 (0.79–1.03)	0.133	0.99
	Sometimes	0.57 (0.48–0.68)	<0.001	<0.001	0.56 (0.48–0.65)	<0.001	<0.001	0.53 (0.43–0.67)	<0.001	<0.001	0.76 (0.59–1.00)	0.045	0.99	0.52 (0.45–0.59)	<0.001	<0.001
	Often	0.27 (0.22–0.34)	<0.001	<0.001	0.34 (0.29–0.41)	<0.001	<0.001	0.29 (0.22–0.37)	<0.001	<0.001	0.37 (0.27–0.50)	<0.001	<0.001	0.26 (0.22–0.30)	<0.001	<0.001
	Very often	0.24 (0.18–0.31)	<0.001	<0.001	0.31 (0.26–0.38)	<0.001	<0.001	0.28 (0.20–0.38)	<0.001	<0.001	0.31 (0.21–0.45)	<0.001	<0.001	0.21 (0.17–0.25)	<0.001	<0.001
Pharmacy	Never	Ref.			Ref.			Ref.			Ref.			Ref.		
	Rarely	1.05 (0.80–1.40)	0.738	0.99	0.96 (0.77–1.21)	0.721	0.99	0.73 (0.53–1.02)	0.060	0.99	0.68 (0.48–0.99)	0.040	0.99	1.19 (0.95–1.49)	0.128	0.99
	Sometimes	0.93 (0.73–1.21)	0.601	0.99	0.65 (0.53–0.81)	<0.001	0.005	0.73 (0.55–0.97)	0.028	0.99	0.67 (0.49–0.93)	0.013	0.99	0.92 (0.76–1.14)	0.440	0.99
	Often	0.56 (0.43–0.73)	<0.001	0.001	0.43 (0.35–0.53)	<0.001	<0.001	0.37 (0.28–0.49)	<0.001	<0.001	0.43 (0.31–0.59)	<0.001	<0.001	0.63 (0.51–0.77)	<0.001	0.001
	Very often	0.37 (0.28–0.49)	<0.001	<0.001	0.34 (0.28–0.43)	<0.001	<0.001	0.26 (0.19–0.36)	<0.001	<0.001	0.22 (0.15–0.31)	<0.001	<0.001	0.45 (0.37–0.56)	<0.001	<0.001
Healthcare contact	Very difficult	Ref.			Ref.			Ref.			Ref.			Ref.		
	Somewhat difficult	0.94 (0.75–1.18)	0.581	0.99	0.65 (0.55–0.76)	<0.001	<0.001	0.58 (0.46–0.74)	<0.001	0.001	0.76 (0.58–1.02)	0.062	0.99	0.94 (0.79–1.11)	0.450	0.99
	Neither easy nor difficult	1.27 (1.04–1.57)	0.024	0.99	0.77 (0.67–0.90)	0.001	0.062	0.74 (0.60–0.93)	0.007	0.674	0.84 (0.65–1.11)	0.204	0.99	1.42 (1.21–1.67)	<0.001	0.002
	Somewhat easy	1.28 (1.04–1.60)	0.023	0.99	0.61 (0.52–0.72)	<0.001	<0.001	0.52 (0.41–0.67)	<0.001	<0.001	0.88 (0.67–1.16)	0.348	0.99	1.46 (1.24–1.73)	<0.001	0.001

	Very easy	1.23 (0.96–1.58)	0.099	0.99	0.50 (0.41–0.61)	<0.001	<0.001	0.62 (0.47–0.82)	0.001	0.090	0.66 (0.46–0.93)	0.018	0.99	1.40 (1.16–1.69)	0.001	0.050
Covid19 vax	Yes vs No	1.84 (1.50–2.28)	<0.001	<0.001	0.12 (0.11–0.13)	<0.001	<0.001	0.05 (0.04–0.05)	<0.001	<0.001	0.27 (0.23–0.32)	<0.001	<0.001	2.23 (1.90–2.64)	<0.001	<0.001

Reason 1 = Getting the flu or transmitting it does not matter; Reason 2 = Fear of side effects/vaccine dangerous/afraid of injection; Reason 3 = Against vaccine; Reason 4 = Vaccine not effective; Reason 5 = All other and no reason given

OR = Odds Ratio; 95%CI: 95% confidence interval; p raw = unadjusted p value; p Bonf = Bonferroni-adjusted p-value

Scholarity = level of education; MMG = frequency of contact with the general practitioner; Pharmacy = frequency of pharmacy visits, Healthcare contact = ease of healthcare access; Covid19 vax = receipt of COVID-19 vaccination; Flu vax 22/23 = receipt of influenza vaccination in the 2022/2023 season, factor not included as not estimable
